# Exposure to Free and Conjugated Forms of Bisphenol A and Triclosan among Pregnant Women in the MIREC Cohort

**DOI:** 10.1289/ehp.1408187

**Published:** 2014-11-21

**Authors:** Tye E. Arbuckle, Leonora Marro, Karelyn Davis, Mandy Fisher, Pierre Ayotte, Patrick Bélanger, Pierre Dumas, Alain LeBlanc, René Bérubé, Éric Gaudreau, Gilles Provencher, Elaine M. Faustman, Eric Vigoren, Adrienne S. Ettinger, Michael Dellarco, Susan MacPherson, William D. Fraser

**Affiliations:** 1 Population Studies Division, Healthy Environments and Consumer Safety Branch, Health Canada, Ottawa, Ontario, Canada; 2 Laboratoire de toxicologie, Institut national de santé publique du Québec, Québec, Quebec, Canada; 3 Population Health and Optimal Health Practices Research Unit, CHU (Centre hospitalier universitaire) de Québec Research Centre, Québec, Quebec, Canada; 4 Institute for Risk Analysis and Risk Communication, Department of Environmental and Occupational Health Sciences, University of Washington, Seattle, Washington, USA; 5 Center for Perinatal, Pediatric and Environmental Epidemiology, Yale School of Public Health, New Haven, Connecticut, USA; 6 The National Children’s Study, *Eunice Kennedy Shriver* National Institute of Child Health and Human Development, National Institutes of Health, Department of Health and Human Services, Bethesda, Maryland, USA; 7 CHU Sainte-Justine Research Center, Mother and Child University Hospital Center, Montreal, Quebec, Canada

## Abstract

**Background:**

Bisphenol A (BPA) and triclosan (TCS) are two nonpersistent chemicals that have been frequently measured in spot urine samples from the general population but less so in pregnant women; however, data are limited on the free (bioactive) and conjugated forms of these phenols.

**Objectives:**

The Maternal-Infant Research on Environmental Chemicals (MIREC) Study addressed these data gaps by utilizing stored maternal urine samples from a large multicenter cohort study of Canadian pregnant women.

**Methods:**

Concentrations of free and conjugated forms of BPA and TCS were measured in about 1,890 first-trimester urine samples by ultra performance liquid chromatograpy–tandem mass spectrometry using isotope dilution.

**Results:**

The glucuronides of BPA and TCS were the predominant forms of these chemicals measured (detected in 95% and 99% of samples, respectively), whereas the free forms were detected in 43% and 80% of samples, respectively. The geometric mean urinary concentrations for glucuronides of BPA and TCS were 0.80 μg/L (95% CI: 0.75, 0.85) and 12.30 μg/L (95% CI: 11.08, 13.65), respectively. Significant predictors of BPA included maternal age < 25 vs. ≥ 35 years, current smoking, low vs. high household income, and low vs. high education. For TCS, urinary concentrations were significantly higher in women ≥ 25 years of age, never vs. current smokers, and women with high household income and high education.

**Conclusions:**

The results from this study represent the largest national-level data on urinary concentrations of free and conjugated forms of BPA and TCS in pregnant women and suggest that maternal characteristics predicting elevated urinary concentrations of these phenols largely act in opposite directions.

**Citation:**

Arbuckle TE, Marro L, Davis K, Fisher M, Ayotte P, Bélanger P, Dumas P, LeBlanc A, Bérubé R, Gaudreau É, Provencher G, Faustman EM, Vigoren E, Ettinger AS, Dellarco M, MacPherson S, Fraser WD. 2015. Exposure to free and conjugated forms of bisphenol A and triclosan among pregnant women in the MIREC cohort. Environ Health Perspect 123:277–284; http://dx.doi.org/10.1289/ehp.1408187

## Introduction

Leaching of bisphenol A (BPA) has been reported from food cans and polycarbonate bottles, paper receipts, and dental sealants and fillings (Hoyle and Budway 1997; Joskow et al. 2006; Lu et al. 2013). BPA does not bioaccumulate and has a very short half-life in humans, with elimination of the conjugated BPA in about 6 hr in the urine (Völkel et al. 2002). Orally administered BPA is rapidly and efficiently absorbed from the gastrointestinal tract and undergoes first-pass metabolism in the gut wall and liver, biotransforming BPA to its conjugated forms: BPA glucuronide (BPAG), which is devoid of estrogenic activity (Matthews et al. 2001) and BPA monosulfate (BPAS) and disulfate (BPADS).

Exposure to BPA is widespread, with > 90% of the populations of the United States and Canada having detectable urinary concentrations (Calafat et al. 2008a; Health Canada 2013). Evidence regarding effects of prenatal BPA exposure on fetal growth and birth weight is conflicting. For example, in a Dutch cohort study, BPA exposure was associated with lower fetal growth rates and lower birth weight (Snijder et al. 2013); however, BPA was not associated with birth weight in a Chinese cohort (Tang et al. 2013). Similarly, some studies have estimated significant associations between maternal urinary concentrations of BPA and child behavior (Braun et al. 2011; Harley et al. 2013; Perera et al. 2012), whereas others have reported no associations (Miodovnik et al. 2011; Yolton et al. 2011). These ambiguous findings may reflect differences in study populations or methodological issues related to exposure assessment.

Triclosan (TCS) is an antibacterial compound used in some cosmetic products, toothpaste, treated textiles, and food contact materials, such as cutting boards and countertops (Health Canada and Environment Canada 2012). TCS may be an endocrine disruptor, with some evidence in laboratory animals of effects on thyroid hormone homeostasis and possibly the reproductive axis (Dann and Hontela 2011). Only two epidemiologic studies have explored potential health effects of prenatal exposure to TCS on birth size, and both reported no significant association (Philippat et al. 2012; Wolff et al. 2008).

Triclosan is highly lipid soluble and rapidly absorbed from the gut, and it has a urinary elimination half-life of 11 hr, with an estimated 0.5% present in the unconjugated form within 24 hr of exposure and the majority of the compound as the glucuronide (Sandborgh-Englund et al. 2006). TCS was detected in approximately three-fourths of the urine samples collected as part of the 2003–2004 National Health and Nutrition Examination Survey (NHANES) of the U.S. population (Calafat et al. 2008b) and the 2009–2011 Canadian Health Measures Survey (Health Canada 2013).

Given the unique vulnerability of pregnant women and their fetuses and the possibility that these chemicals may be endocrine disruptors, it is important to examine the extent of exposure in this population, especially during the critical exposure window of the first trimester. Because the free form of these phenols may be more toxicologically active than the conjugated forms, measurement of urinary concentrations of free BPA and TCS may provide a superior metric of the biologically effective dose.

The objectives of the current study were to *a*) measure the extent of exposure to free and conjugated forms of BPA and TCS during pregnancy among a population of Canadian women; and *b*) identify predictors of elevated body burdens of these chemicals. This research offered a unique opportunity to efficiently examine these issues in a large diverse population using stored biological specimens and data collected in this prospective pregnancy cohort study.

## Methods

*Participants*. The Maternal-Infant Research on Environmental Chemicals (MIREC) Study is a national-level pregnancy cohort of approximately 2,000 women recruited in the first trimester of pregnancy from 10 cities across Canada between 2008 and 2011 (Arbuckle et al. 2013). The protocol was approved by ethics committees at Health Canada and the Sainte-Justine University Hospital Center; study subjects gave written informed consent.

*Data collection*. We collected detailed information on demographic and lifestyle factors from questionnaires administered at recruitment in the first trimester. The date and time of the urine collection, as well as the time since last urine void, were also noted.

*Urine collection and field blanks*. During the first-trimester clinic visit, a spot urine sample was collected in polypropylene cups, aliquoted into 30-mL Nalgene® tubes, frozen at –20°C, and shipped on dry ice to the MIREC Biobank. Of the 2,001 participants, 43 did not consent to the Biobank, 18 women subsequently withdrew from the study, 47 urine samples were not collected, and 3 samples were insufficient, leaving a total of 1,890 urine samples for analysis.

We included field blanks to assess the potential contamination from the material used for collection and storage of urine samples as well as from the environment of collection sites. Water (Steril.O reagent-grade deionized distilled water) was used as a surrogate matrix for urine during the process. Water was poured into polypropylene cups and transferred to polypropylene storage tubes using the same material as for urine samples. Water samples were analyzed following pentafluorobenzyl bromide derivatization by gas chromatography–tandem mass spectrophotometry (GC-MS/MS) using the method previously developed in our laboratory and described by Provencher et al. (2014). Results showed that all field blanks were free of BPA and TCS contamination. All materials in contact with urine samples had been prescreened and found not to be a source of contamination.

*Analytical methods*. The liquid chromatography (LC)-MS/MS methods for the analysis of free and conjugated forms of BPA and TCS in urine were described previously by Provencher et al. (2014). Briefly, free BPA and TCS and their isotope-labeled standards, ^13^C_12_-BPA and ^13^C_12_-TCS, were derivatized with dansyl chloride directly in 1 mL of urine. A liquid–liquid extraction with hexane was subsequently performed and the organic phase evaporated prior to reconstitution in a solution of acetonitrile:H_2_O (50:50, vol:vol). The LC-MS/MS (UPLC Acquity and Xevo TQ-S; Waters) was operated in electrospray positive and multiple reaction monitoring mode. Chromatographic separation was achieved on an Acquity UPLC HSS T3, 1.8 μm, 50 × 2.1 mm analytical column (Waters) using a mobile phase gradient with 0.1% aqueous formic acid solution and acetonitrile.

The conjugated metabolites BPAS, BPADS, BPAG, TCSS (TCS sulfate), and TCSG (TCS glucuronide) and their isotope-labeled standards BPAS-*d_6_*, BPADS-*d_6_*, BPAG-*d_6_*, TCSS-*d_3_*, and TCSG-*d_3_* were extracted from 1.5 mL of urine by solid phase extraction using a weak anion exchange phase (Strata X-AW; Phenomenex). Analytes were eluted from the cartridge using a solution of 1% ammonium hydroxide (NH_4_OH) in methanol. The extracts were evaporated to dryness and reconstituted in a solution of 25% methanol in water. The same LC-MS/MS instrument and analytical column were used as for the free species, but the MS/MS was operated in the electrospray-negative and multiple reaction monitoring mode. A mobile phase gradient from aqueous NH_4_OH (2%) to an NH_4_OH–methanol solution (0.1%) was used to obtain proper chromatographic resolution of conjugated compounds.

*Laboratory quality control (QC)*. Several QC samples, reagents blanks, and urine blanks were incorporated into each batch of samples. In-house reference materials were prepared by spiking human urine to yield low (0.18 μg BPA/L and 0.9 μg TCS/L) and high (1.5 μg BPA/L and 7.5 μg TCS/L) concentrations. For conjugated species, human urine was spiked to obtained reference materials at three different concentration levels: low (0.2 μg/L), medium (2 μg/L for sulfate metabolites; 3 μg/L for glucuronide metabolites), and high (15 μg/L for sulfate metabolites; 60 μg/L for glucuronide metabolites). The intraday precision varied from 2.5% to 7.7%, and the interday precision ranged from 4.3% to 13% depending on the analyte. The accuracy was –3.7% for free BPA and –1.0% for free TCS. The accuracy for conjugated forms ranged from –2.1% to 13.3% depending on the analyte. Detailed quality assurance/QC procedures were described by Provencher et al. (2014).

*Statistical analysis*. Two different approaches were used to calculate summary statistics for the biomonitoring results that were below the limits of detection (LODs). The first approach used values generated by the laboratory instrument, and observations that were reported as zero were replaced by one-half the next smallest value (other than zero) for that contaminant. In the second approach, censoring methods were used by applying survival analysis techniques to left-censored data that have been demonstrated by other authors (Helsel 2012; Nysen et al. 2012) to improve estimation and reduce bias. To account for nondetects, the geometric mean (GM) from a lognormal random variable with censoring was calculated using the maximum likelihood method (MLE) and compared with the empirical median from the Kaplan-Meier approach. The Greenwood estimate of variance was used for Kaplan-Meier confidence intervals. We report summary statistics for both the unadjusted and specific gravity (SG)–adjusted contaminants.

In order to compare concentrations of free and conjugated forms of BPA or TCS, we expressed the concentrations of glucuronides and sulfates as BPA (or TCS) equivalents. Total BPA or TCS was calculated by summing the free and conjugated forms, and the most conservative LOD of the components was assigned to the total to determine the percentage below the LOD.

We calculated GM urinary concentrations for each level of potential predictive variables for all analytes that had at least 50% of the data above the LOD in all groups (as justified by Helsel 2012). For analysis of the associations between potential predictors and the urinary metabolite, SG was included as a covariate in the regression model using analysis of covariance (ANCOVA) (Kutner et al. 2005). ANCOVA adjusts the mean values compared in each level of the potential predictor such that the levels are compared at the same value of the covariate (in this case, SG). The assumptions of ANCOVA are similar to those of analysis of variance (normality and constant variance of residuals) with an additional assumption, that is, the slopes of the relationship between the covariate (SG) and the urinary metabolite must be similar in each level of the potential predictor. The assumptions of normality and equal variance of residuals were tested using the Anderson Darling test and Levene’s test, respectively. The assumption of equal slopes between levels of the potential predictor and the covariate is crucial for ANCOVA to be valid. This reduces to testing the interaction between the potential predictor and SG. When the assumption of equal slopes is not validated (*p* < 0.05), separate treatment regression lines need to be estimated and then compared (Kutner et al. 2005). This implies fitting the ANCOVA model with the interaction between the potential predictor and SG and then comparing the means of the urinary metabolite in each of the groups of the potential predictor at the 25th, 50th, and 75th percentiles of the covariate SG. When the assumptions of normality and constant variance (for the ANCOVA model) were not satisfied, nonparametric methods were applied. Essentially this involved running the models on the ranks of the data. When the overall *F*-test for group differences of the potential predictor from ANCOVA models was significant (*p* < 0.05), pairwise comparisons were carried out using the Scheffé correction for multiple comparisons to determine significant group differences. This correction ensures that the overall false-positive rate from multiple comparisons is < 0.05.

Statistical analysis was performed using SAS Enterprise Guide 4.2 (SAS Institute Inc.) and R (R Development Core Team). For the censoring methods, we used functions from the R libraries NADA and SURVIVAL. Unless otherwise indicated, a 5% significance level (α = 0.05) was implemented throughout.

## Results

*Participant characteristics*. Our participants tended to be well-educated, > 30 years of age, and born in Canada, and they had never smoked, had an underweight or normal body mass index (BMI) prior to pregnancy, and had a parity of 0 or 1 (see Supplemental Material, Table S1).

*BPA*. Detectable urinary concentrations of free BPA, BPAG, and BPAS were found in 43.24%, 94.75%, and 23.43% of the samples, respectively (Table 1). We did not detect BPADS in any samples. The GM urinary concentration for BPAG using the machine readings was 0.80 μg/L [95% confidence interval (CI): 0.75, 0.85], similar to the result using the censoring methods (see Supplemental Material, Table S2). The GM of the ratio of free BPA to total BPA was 1.01%, and the ratio of BPAG to total BPA was 90.27% (data not shown).

**Table 1:** Summary of first trimester urinary concentrations of BPA (bisphenol A) and TCS (triclosan), volumetric- and specific gravity (SG)-adjusted (μg equivalents/L).

Contaminant	*n*	LOD	Percent < LOD	Minimum	Machine readings^*a *^				
					GM (95% CI)	5th percentile	Median	95th percentile	Maximum
BPA disulfate (BPADS)	1,890	0.47	100.0	ND	0.01 (0.01, 0.01)	0.01	0.01	0.01	0.36
SG-adjusted	1,887			ND	0.01 (0.01, 0.01)	0.00	0.01	0.04	0.61
BPA glucuronide (BPAG)	1,889	0.11	5.2	ND	0.80 (0.75, 0.85)	0.11	0.84	6.07	136.15
SG-adjusted	1,886			ND	0.91 (0.86, 0.95)	0.24	0.87	4.65	252.86
Free BPA	1,885	0.012	56.8	ND	0.01 (0.01, 0.01)	0.00	0.01	0.15	2.82
SG-adjusted	1,882			ND	0.01 (0.01, 0.01)	0.00	0.01	0.15	2.52
BPA monosulfate (BPAS)	1,885	0.03	76.6	ND	0.00 (0.00, 0.00)	0.00	0.01	0.12	1.79
SG-adjusted	1,882			ND	0.00 (0.00, 0.00)	0.00	0.01	0.11	2.25
Total BPA	1,879	0.47	30.8	ND	0.88 (0.83, 0.93)	0.13	0.89	6.19	137.82
SG-adjusted	1,876			ND	1.00 (0.96, 1.04)	0.29	0.92	4.78	255.95
Total TCS	1,861	0.12	0.6	ND	12.64 (11.38, 14.03)	0.43	8.74	697.58	6784.3
SG-adjusted	1,858			ND	14.36 (13.01, 15.85)	0.72	9.18	571.10	4199.8
TCS glucuronide (TCSG)	1,868	0.12	0.6	ND	12.30 (11.08, 13.65)	0.43	8.42	676.47	6760.29
SG-adjusted	1,865			ND	13.94 (12.63, 15.40)	0.70	9.03	559.97	4184.94
Free TCS	1,882	0.008	20.0	ND	0.06 (0.05, 0.07)	0.00	0.06	7.70	784.47
SG-adjusted	1,879			ND	0.07 (0.06, 0.08)	0.00	0.06	5.74	329.34
TCS sulfate (TCSS)	1,890	0.02	85.3	ND	0.00 (0.00, 0.00)	0.00	0.00	0.08	4.26
SG-adjusted	1,887			ND	0.00 (0.00, 0.00)	0.00	0.00	0.06	3.69
Free/total BPA (%)	1,879		56.8	0.0006	1.01 (0.93, 1.10)	0.03	1.18	12.25	63.47
Free/total TCS (%)	1,861		20.0	0.0006	0.50 (0.46, 0.54)	0.01	0.58	6.17	82.5
ND, estimate below the laboratory’s LOD. SG missing for 3 samples. ^***a***^Some statistics are below the laboratory method’s LOD and should be interpreted with caution.									

The log of BPAG was positively correlated with the log of free BPA (*r* = 0.38, *p* < 0.0001) and the log of total BPA (*r* = 0.98, *p* < 0.0001) (data not shown). The log of free BPA was positively correlated with the log of total BPA (*r* = 0.42, *p* < 0.0001) (see Supplemental Material, Figure S1a). As log total urinary BPA increased, free BPA also increased and the ratio of free to total BPA decreased (*r* = –0.22, *p* < 0.0001) (see Supplemental Material, Figure S1b).

In the analysis of characteristics associated with urinary concentrations, BPAG (Table 2) and total BPA concentrations (see Supplemental Material, Table S3) were significantly higher in urine samples from women < 25 years of age compared with women ≥ 35, from smokers compared with former or never smokers, and from women with lower household income (< $50,000 vs. > $100,000) and lower education (≤ high school vs. ≥ university degree). We observed a significant interaction between SG and time of urine collection; for example, at the 25th and 50th percentiles of SG, urine samples collected after 1500 hours had significantly higher BPAG and total BPA than samples collected earlier in the day. However, at the 75th percentile of SG, adjusted GM concentrations of BPAG and total BPA were similar with respect to time of urine collection (Table 3; see also Supplemental Material, Table S4).

**Table 2:** Predictors of maternal urinary concentrations of free and conjugated BPA (reported as μg BPA equivalents/L) with specific gravity as a covariate.

Characteristic	*n*	BPAG				Free BPA			
		Percent < LOD	*p*-Value^*a*^	Pairwise^*b*^	GM (95% CI)^*c*^	Percent < LOD	*p*-Value^*a*^	Pairwise^*b*^	GM (95% CI)^*c*^
Maternal age (years)
< 25	125	0.80	0.0013	A	1.06 (0.88, 1.28)	39.52	< 0.0001	A	0.02 (ND, 0.02)
25–29	441	4.76		AB	0.83 (0.75, 0.91)	52.50		AB	ND (ND, ND)
30–34	682	5.43		AB	0.83 (0.76, 0.90)	57.27		B	ND (ND, ND)
≥ 35	641	6.24		B	0.72 (0.66, 0.78)	62.50		C	ND (ND, ND)
Parity
0	834	4.68	0.0816		0.85 (0.79, 0.92)	55.10	0.0774^*d*^		ND (ND, ND)
1	765	6.27			0.78 (0.72, 0.84)	58.19			ND (ND, ND)
≥ 2	288	4.17			0.73 (0.65, 0.83)	57.49			ND (ND, ND)
Maternal smoking status
Current/quit during pregnancy	225	3.11	< 0.0001^*d*^	A	1.01 (0.88, 1.16)	45.33	0.0056	A	0.01 (ND, 0.02)
Former	521	4.03		B	0.82 (0.75, 0.90)	59.92		B	ND (ND, ND)
Never	1,141	6.22		B	0.76 (0.71, 0.81)	57.59		B	ND (ND, ND)
Maternal education
High school or less	168	4.17	0.0068^*d*^	A	0.90 (0.76, 1.06)	42.86	< 0.0001^*d*^	A	0.01 (ND, 0.02)
College courses or diploma	540	4.63		AB	0.83 (0.75, 0.90)	51.49		A	ND (ND, ND)
University degree	1,179	5.68		B	0.78 (0.73, 0.83)	61.09		B	ND (ND, ND)
Place of birth
Elsewhere	353	6.80	0.0029^*d*^	A	0.70 (0.63, 0.79)	60.23	0.5268		ND (ND, ND)
Canada	1,536	4.88		B	0.83 (0.78, 0.87)	55.97			ND (ND, ND)
Prepregnancy BMI (kg/m^2^)
< 25 (underweight–normal)	1,108	5.60	0.2206^*d*^		0.78 (0.73, 0.83)	62.90	< 0.0001	A	ND (ND, ND)
25–29 (overweight)	384	6.25			0.81 (0.73, 0.90)	50.91		B	ND (ND, ND)
≥ 30 (obese)	261	3.83			0.84 (0.73, 0.96)	42.53		B	0.01 (ND, 0.02)
Household income
≤ $50,000	326	3.68	0.0045^*d*^	A	0.89 (0.79, 1.01)	45.23	< 0.0001	A	0.01 (ND, 0.02)
$50,001–100,000	754	5.17		AB	0.82 (0.76, 0.88)	55.38		B	ND (ND, ND)
> $100,000	726	5.79		B	0.76 (0.70, 0.82)	63.90		C	ND (ND, ND)
Season urine collected
Fall	547	4.94	0.0720^*d*^		0.80 (0.73, 0.87)	60.81	0.0002	A	ND (ND, ND)
Winter	456	4.17			0.84 (0.76, 0.93)	61.95		A	ND (ND, ND)
Spring	442	7.01			0.73 (0.66, 0.81)	53.17		B	ND (ND, ND)
Summer	444	4.95			0.85 (0.76, 0.93)	50.11		B	ND (ND, ND)
Fasting status
No	1,827	5.36	0.7425		0.80 (0.76, 0.84)	57.05	0.0037	A	ND (ND, ND)
Yes	37	0.00			0.85 (0.60, 1.20)	45.95		B	0.02 (ND, 0.04)
Time since last urination (min)
≤ 75	488	7.58	0.0020	A	0.76 (0.69, 0.84)	65.23	< 0.0001^*d*^	A	ND (ND, ND)
76–120	596	6.21		A	0.74 (0.67, 0.80)	59.26		AB	ND (ND, ND)
121–170	266	2.26		B	0.98 (0.86, 1.11)	51.50		BC	ND (ND, ND)
> 170	446	2.69		AB	0.85 (0.77, 0.94)	45.29		C	0.01 (0.01, 0.02)
ND, estimate below the laboratory’s LOD. ^***a***^*p*-Value for overall group effect based on machine readings. ^***b***^Pairwise comparisons were made only when the overall group effect was significant (*p *< 0.05). Group levels with the same letter are not significantly different by Scheffé multiple comparisons; group levels with different letters are significantly different. ^***c***^95% CIs for the GMs were corrected for multiple comparisons using Scheffé correction. ^***d***^ANCOVA model based on the ranks of the data (nonparametric ANCOVA); each group was assumed to have the same slope with respect to the covariate [i.e., the interaction between the potential predictor (characteristic) and specific gravity was not significant (*p* > 0.05)].									

**Table 3:** Comparisons of maternal urinary concentrations of free and conjugated BPA (reported as μg BPA equivalents/L) at the various times of urine collection with respect to different levels of specific gravity (SG).

Chemical	Time of urine collection (hours)	P25 of SG^*a*^		P50 of SG^*b *^		P75 of SG^*c *^	
		Differences	Adjusted GM (95% CI)	Differences	Adjusted GM (95% CI)	Differences	Adjusted GM (95% CI)
BPAG^*d*^	0600–0900	AB	0.27 (0.15, 0.48)	AB	0.62 (0.41, 0.94)		1.65 (1.03, 2.62)
	0900–1200	A	0.34 (0.31, 0.37)	A	0.66 (0.62, 0.71)		1.44 (1.30, 1.60)
	1200–1500	A	0.34 (0.30, 0.38)	A	0.69 (0.63, 0.75)		1.56 (1.41, 1.73)
	1500–1800	B	0.57 (0.48, 0.67)	B	0.94 (0.84, 1.06)		1.72 (1.52, 1.94)
	1800–2400	AB	0.73 (0.41, 1.29)	AB	1.21 (0.81, 1.80)		2.18 (1.51, 3.15)
Free BPA^*d*^	0600–0900		0.01 (0.00, 0.01)	AB	0.01 (0.00, 0.01)	ABC	0.01 (0.00, 0.02)
	0900–1200		0.01 (0.01, 0.01)	A	0.01 (0.01, 0.01)	A	0.02 (0.01, 0.02)
	1200–1500		0.01 (0.00, 0.01)	B	0.01 (0.01, 0.01)	AB	0.01 (0.01, 0.02)
	1500–1800		0.01 (0.00, 0.01)	B	0.01 (0.01, 0.01)	C	0.01 (0.01, 0.01)
	1800–2400		0.01 (0.00, 0.02)	AB	0.01 (0.00, 0.01)	BC	0.01 (0.00, 0.01)
^***a***^25th percentile of SG = 1.007. ^***b***^50th percentile of SG = 1.013. ^***c***^75th percentile of SG = 1.020. ^***d***^ANCOVA model, assuming separate slopes for each of the collection times because the interaction between the time of urine collection and SG was significant (*p *< 0.05). Pairwise comparisons were made only when the overall difference between the collection times was significant at that level of SG. Therefore, where no pairwise comparisons were made, the collection times were not significantly different.							

*Triclosan*. Most of the women had detectable concentrations of total TCS, TCSG, and free TCS (Table 1). The GM urinary concentrations of total TCS and TCSG were 12.64 μg/L (95% CI: 11.38, 14.03) and 12.30 μg/L (95% CI: 11.08, 13.65), respectively. The GM of the ratio of free TCS to total TCS was 0.50%, and that for the ratio of TCSG to total TCS was 97.82% (data not shown).

All of the log-transformed TCS metabolites were highly correlated with each other (data not shown). TCSG was correlated with free TCS (*r* = 0.81, *p* < 0.0001) and total TCS (*r* = 1.00, *p* < 0.0001). Similarly, free TCS was correlated with total TCS (*r* = 0.82, *p* < 0.0001).

We observed significantly lower urinary concentrations of total TCS (see Supplemental Material, Table S5) and TCSG (Table 4) in women who were smokers compared with never smokers, were < 25 years of age, did not have a university degree, and had household income ≤ $100,000. Only free TCS varied by time of urine collection, with urine collected between 0900 and 1200 hours significantly higher than that collected between 1500 and 1800 hours. A significant interaction between SG and parity was observed. Specifically at the 25th and 50th percentiles of SG, TCS levels were statistically similar between the different parity groups; however, at the 75th percentile of SG, adjusted GM TCS concentrations were higher for women with parity of 0 than those with parity of 1 (Table 5; see also Supplemental Table S6).

**Table 4:** Predictors of maternal urinary concentrations of free and conjugated TCS (reported as μg TCS equivalents/L) with specific gravity as a covariate

Characteristic	*n*	TCSG				Free TCS			
		Percent < LOD	*p*-Value^*a*^	Pairwise^*b*^	GM (95% CI)^*c*^	Percent < LOD	*p*-Value^*a*^	Pairwise^*b*^	GM (95% CI)^*c*^
Maternal age (years)
< 25	122	0.81	< 0.0001^*d*^	A	5.11 (3.48, 7.51)	17.89	0.1173		0.04 (0.02, 0.06)
25–29	437	0.23		B	13.28 (10.83, 16.29)	17.50			0.07 (0.06, 0.09)
30–34	672	0.89		B	13.08 (11.09, 15.42)	20.67			0.07 (0.05, 0.08)
≥ 35	630	0.47		B	12.93 (10.91, 15.33)	21.51			0.06 (0.05, 0.07)
Maternal smoking status
Current/quit during pregnancy	220	0.45	0.0030	A	8.55 (6.41, 11.42)	20.09	0.0236^*d*^	AB	0.05 (0.03, 0.07)
Former	515	0.97		AB	10.82 (8.96, 13.06)	22.59		A	0.05 (0.04, 0.06)
Never	1,124	0.44		B	13.97 (12.30, 15.87)	18.89		B	0.07 (0.06, 0.09)
Maternal education
High school or less	163	0.60	0.0001	A	7.34 (5.25, 10.28)	13.94	0.1232^*d*^		0.05 (0.04, 0.08)
College courses or diploma	530	0.38		A	10.31 (8.57, 12.41)	22.72			0.06 (0.04, 0.07)
University degree	1,166	0.69		B	14.33 (12.64, 16.24)	19.61			0.07 (0.06, 0.08)
Place of birth
Elsewhere	347	0.86	0.3025		11.01 (8.75, 13.86)	21.02	0.8749^*d*^		0.06 (0.05, 0.08)
Canada	1,514	0.53			12.60 (11.28, 14.06)	19.80			0.06 (0.05, 0.07)
Prepregnancy BMI (kg/m^2^)
< 25 (underweight-normal)	1,089	0.55	0.7914^*d*^		11.80 (10.37, 13.43)	22.76	0.0002^*d*^	A	0.05 (0.04, 0.06)
25–29 (overweight)	383	0.26			12.29 (9.88, 15.29)	19.79		AB	0.07 (0.05, 0.09)
≥ 30 (obese)	258	0.39			12.44 (9.52, 16.26)	10.38		B	0.11 (0.08, 0.15)
Household income
≤ $50,000	318	0.31	< 0.0001	A	9.27 (7.31, 11.76)	16.51	0.1950		0.06 (0.05, 0.09)
$50,001–100,000	743	0.40		A	10.69 (9.15, 12.50)	20.61			0.05 (0.05, 0.07)
> $100,000	717	0.84		B	16.07 (13.70, 18.85)	21.10			0.07 (0.06, 0.09)
Season urine collected
Fall	540	0.19	0.3043	A	14.16 (11.77, 17.04)	20.66	0.6682^*d*^		0.06 (0.05, 0.08)
Winter	448	0.44		A	12.24 (10.00, 14.98)	21.41			0.05 (0.04, 0.07)
Spring	436	0.91		B	11.21 (9.14, 13.76)	20.09			0.07 (0.05, 0.09)
Summer	437	0.92		B	11.34 (9.23, 13.92)	17.79			0.07 (0.05, 0.09)
Fasting status
No	1,799	0.61	0.2239		12.27 (11.09, 13.57)	20.38	0.6824^*d*^		0.06 (0.05, 0.07)
Yes	37	0.00			7.88 (3.89, 15.97)	8.11			0.08 (0.03, 0.19)
Time of urine collection (hours)
0600–0900	28	3.57	0.4257		13.71 (6.09, 30.85)	14.29	0.0002^*d*^	AB	0.09 (0.03, 0.24)
0900–1200	797	0.63			13.66 (11.73, 15.92)	20.25		A	0.09 (0.07, 0.10)
1200–1500	635	0.63			11.68 (9.85, 13.85)	20.28		AB	0.06 (0.05, 0.07)
1500–1800	364	0.27			10.83 (8.64, 13.57)	20.22		B	0.04 (0.03, 0.05)
1800–2400	35	0.00			9.62 (4.70, 19.70)	11.43		AB	0.04 (0.01, 0.09)
Time since last urination (min)
≤ 75	480	1.04	0.1182		10.20 (8.38, 12.40)	27.72	< 0.0001	A	0.04 (0.03, 0.05)
76–120	593	0.67			13.34 (11.19, 15.90)	21.48		B	0.07 (0.05, 0.08)
121–170	262	0.38			14.18 (10.88, 18.47)	16.23		B	0.07 (0.05, 0.10)
> 170	438	0.00			13.28 (10.82, 16.31)	10.61		B	0.10 (0.07, 0.12)
^***a***^*p*-Value for overall group effect based on machine readings. ^***b***^Pairwise comparisons were made only when the overall group effect was significant (*p *< 0.05). Group levels with the same letter are not significantly different by Scheffé multiple comparisons; group levels with different letters are significantly different. ^***c***^The 95% CIs for the GMs were corrected for multiple comparisons using Scheffé correction. ^***d***^ANCOVA model based on the ranks of the data (nonparametric ANCOVA); each group was assumed to have the same slope with respect to the covariate [i.e., the interaction between the potential predictor (characteristic) and specific gravity was not significant (*p* > 0.05)].									

**Table 5:** Comparisons of maternal urinary concentrations of free and conjugated TCS (reported as μg TCS equivalents/L) at the various parity groups with respect to different levels of specific gravity (SG).

Chemical	Parity	P25 of SG^*a*^		P50 of SG^*b *^		P75 of SG^*c *^	
		Differences	Adjusted GM (95% CI)	Differences	Adjusted GM (95% CI)	Differences	Adjusted GM (95% CI)
TCSG^*d *^	0	—	6.25 (5.13, 7.60)	—	12.30 (10.60, 14.28)	A	27.13 (22.20, 33.14)
	1	—	6.98 (5.64, 8.63)	—	10.93 (9.33, 12.80)	B	18.43 (15.22, 22.32)
	≥ 2	—	5.15 (3.61, 7.35)	—	9.53 (7.35, 12.37)	AB	19.53 (14.30, 26.67)
Free TCS^*e *^	0	—	0.02 (0.01, 0.02)	—	0.06 (0.05, 0.07)	A	0.26 (0.20, 0.33)
	1	—	0.02 (0.02, 0.03)	—	0.05 (0.04, 0.06)	B	0.15 (0.12, 0.19)
	≥ 2	—	0.02 (0.01, 0.02)	—	0.04 (0.03, 0.06)	B	0.16 (0.11, 0.23)
^***a***^25th percentile of SG = 1.007. ^***b***^50th percentile of SG = 1.013. ^***b***^75th percentile of SG = 1.020. ^***d***^ANCOVA model, assuming separate slopes for each of the parity groups because the interaction between parity and SG was significant (*p *< 0.05). Pairwise comparisons were made only when the overall difference between the parity groups was significant at that level of SG. Therefore, where no pairwise comparisons were made, the parity groups were not significantly different. ^***e***^ANCOVA model based on the ranks of the data (nonparametric model), assuming separate slopes for each of the parity groups because the interaction between parity and SG was significant (*p *< 0.05). Pairwise comparisons were made only when the overall difference between the parity groups was significant at that level of SG. Therefore, where no pairwise comparisons were made, the parity groups were not significantly different.							

Comparing the two methods for dealing with values < LOD, the median from the Kaplan-Meier was similar to the median based on machine readings, and the GM from the censored MLE compared well with the GM based on machine readings (Table 1; see also Supplemental Material, Table S2). Overall, *p*-values for the censoring methods were similar to machine readings, but were generally more conservative (data not shown).

## Discussion

Given the potential health concerns of prenatal exposure to BPA and TCS on the developing infant and on the physiological and behavioral changes during pregnancy that can affect exposure to and disposition of chemicals in the body (Moya et al. 2014), it is critical to have biomonitoring data on a large, diverse population of pregnant women for the critical window of development. Furthermore, data on the free and conjugated compounds will contribute to understanding the toxicokinetics and potential health risks of the biologically active compounds.

Total BPA urinary concentrations (unadjusted for urine dilution) have been measured in several cohorts of pregnant women in the United States, with medians ranging from 1.0 to 2.0 μg/L (Braun et al. 2011; Harley et al. 2013; Hoepner et al. 2013; Mortensen et al. 2014; Wolff et al. 2008), somewhat higher than we observed in the MIREC cohort (0.89 μg/L). Urinary concentrations of BPA in U.S. women ≥ 20 years of age (NHANES 2009–2010) [Centers for Disease Control and Prevention (CDC) 2013] were higher at the 50th (1.8 vs. 0.9 μg/L) and 95th (9.6 vs. 6.2 μg/L) percentiles than in MIREC (2008–2011). A previous comparison of U.S. and Canadian urinary concentrations of BPA in the general population was not able to identify any methodological differences to explain the statistically significant lower levels in Canada (LaKind et al. 2012), which might suggest that there are population differences (e.g., consumer product formulations or avoidance of BPA-containing products) in Canada.

Only one study has reported urinary concentrations of free BPA in pregnant women (Vandentorren et al. 2011); however, contamination of the samples from exogenous sources during collection likely occurred. Free BPA has been measured in the urine of nonpregnant adult populations with widely varying detection rates of ≤ 10% (Fox et al. 2011; Völkel et al. 2008; Ye et al. 2005) to > 70% (Liao and Kannan 2012; Schöringhumer and Cichna-Markl 2007). The comparable figure in our study was 43%.

A few studies have measured the conjugated forms of BPA in adult populations (e.g., Kim et al. 2003; Liao and Kannan 2012; Ye et al. 2005). In a study of 163 subjects in France, Harthé et al. (2012) reported a GM urinary concentration of BPAG of 4.64 μg/L (corresponding to 2.62 μg/L BPA), which represented 79% of urinary total BPA. The GM concentration of BPAG in our study was considerably lower at 0.80 μg/L, with a ratio of BPAG to total BPA of 90%.

There are a number of possible explanations for differences between studies in levels or proportions of free and total BPA, including disparities in laboratory methods and their sensitivity, differences in the study populations, hydrolysis of the conjugates, and contamination of the samples. Although free BPA has been measured in several studies (reviewed by Vandenberg et al. 2010), other authors have argued that the detection of free BPA resulted from contamination of the sample or deconjugation of the BPAG during storage or sample preparation (Dekant and Völkel 2008; Teeguarden et al. 2011). Because derivatization with dansyl chloride was the first step of the analytical procedure for free BPA and TCS determination in the present study, contamination by the free forms of these ubiquitous phenols was prevented during the subsequent steps of sample preparation (Provencher et al. 2014). Furthermore, our in-house reference materials showed no decrease in conjugate concentrations after several months of storage at –20°C, and our field blanks and prescreening of collection materials did not provide any evidence of contamination with BPA. Therefore, we believe that we minimized, as much as possible, potential sources of external contamination that could artifactually inflate urinary concentrations of free BPA in our participants. It should be noted, however, that on average, only about 1% of the total BPA was present in the unconjugated form and that the median free BPA was at the method’s LOD.

There are some inconsistencies among studies that have identified major predictors of urinary BPA concentrations in pregnant women. For example, whereas our study and one in Spain (Casas et al. 2013) found higher BPA concentrations in younger women, studies in Puerto Rico (Meeker et al. 2013) and the United States (Braun et al. 2011; Quirós-Alcalá et al. 2013; Robledo et al. 2013) reported no significant associations with maternal age. Smoking and low education (< 12 years) were significant predictors of higher BPA concentrations in MIREC and in studies in Cincinnati, Ohio (*n* = 388; Braun et al. 2011) and Spain (*n* = 479; Casas et al. 2013), but not in studies in California (*n* = 470; Quirós-Alcalá et al. 2013), New York City (*n* = 568; Hoepner et al. 2013), Puerto Rico (*n* = 105; Meeker et al. 2013), or Korea (*n* = 757; Lee et al. 2014).

Similar to BPA, the predominant TCS metabolite measured in MIREC was the glucuronide form; however, free TCS was detected in about 80% of the urine samples. The median ratio of free to total TCS (0.57%) is comparable to that reported following oral ingestion of TCS in 10 volunteers, where 0.9% of the TCS excreted was reportedly in the free form 24–48 hr after exposure (Sandborgh-Englund et al. 2006).

Several studies have measured total TCS in urine from pregnant women (see Supplemental Material, Table S7). Median urinary concentrations were considerably higher in studies conducted in Puerto Rico (Meeker et al. 2013) and France (Philippat et al. 2012) than in those conducted in California (Biomonitoring California 2013), New York City (Philippat et al. 2013; Wolff et al. 2008), Spain (Casas et al. 2011), or MIREC. Regional and population differences may be related to the use and/or availability of consumer products containing TCS. There was no significant difference between median urinary TCS concentrations in U.S. women in NHANES 2009–2010 (11.1 μg/L; CDC 2013) or Canadian women in the Canadian Health Measures Survey 2009–2011 (16 μg/L; Health Canada 2013) compared with those in MIREC (8.7 μg/L).

Data on major predictors of TCS exposure are limited. In MIREC, urinary TCS concentrations were significantly elevated in women with a university degree (compared with those with less education), > $100,000 compared with ≤ $100,000 household income, ≥ 25 compared with < 25 years of age, and never smokers compared with former or current smokers. In the U.S. general adult population, TCS concentrations were significantly higher in households with income of ≥ $20,000; they appeared to peak in the third decade of life and then decline slowly thereafter (Calafat et al. 2008b). In Puerto Rico, maternal urinary TCS was higher in older pregnant women (> 30 years) but was not associated with maternal education or income (Meeker et al. 2013).

A major concern in conducting biomonitoring studies is potential contamination of biospecimens via materials and processes (Longnecker et al. 2013; Salgueiro-González et al. 2012; Ye et al. 2013), resulting in higher measured concentrations than were actually present. Alternatively, concentrations may be artifactually reduced if, for example, free TCS adheres to the collection containers or other materials (Provencher et al. 2014). In MIREC, the ratio of free to total TCS varied from a minimum of < 1% to 82.5%, indicating that this was likely not a consistent problem. The use of field and laboratory blanks and prescreening of collection materials can assist with identifying potential sources of contamination; we used these QC methods in the present study and found contamination not to be a concern.

Consideration of the ability of a single spot urine sample to predict an individual’s exposure over a period of time is especially important for short-lived chemicals such as BPA and TCS. Previous studies have indicated that although the intraclass correlation coefficient (ICC) for BPA is low (< 0.25) across pregnancy (Braun et al. 2011; Fisher et al. 2014; Meeker et al. 2013; Philippat et al. 2013), the ICC for TCS is higher (> 0.47) (Bertelsen et al. 2014; Meeker et al. 2013; Philippat et al. 2013); the results presented here should be interpreted with this potential limitation of spot urine samples in mind.

## Conclusions

The present study is one of only a few to measure the extent of exposure to free and conjugated forms of BPA and TCS, especially during the critical period of pregnancy. We observed median concentrations that were lower than the estimated biomonitoring equivalents (24-hr average concentrations that are consistent with an existing health-based exposure guideline such as a reference dose or tolerable daily intake) for total BPA (1–2 mg/L; Krishnan et al. 2010a) and total TCS (2.6–6.4 mg/L; Krishnan et al. 2010b) in urine.

Maternal age, household income, education, and smoking status were significant predictors for total BPA and total TCS exposure, however, in opposite directions. The consumer products used by individuals (and thus their exposure to various chemicals) can vary by age and education (Barrett et al. 2014; Biesterbos et al. 2013; Wu et al. 2010) and possibly by culture or ethnicity. These data will be important in assessing potential risks of these chemicals and in developing profiles of exposure, particularly in identifying women with elevated exposures.

## Supplemental Material

(846 KB) PDF
