# Supplementary material for: Exposure to Free and Conjugated Forms of Bisphenol A and Triclosan among Pregnant Women in the MIREC Cohort
Source: Environ Health Perspect. 2014 Nov 21;123(4):277–84. doi: 10.1289/ehp.1408187 (PMC4384201; doi:10.1289/ehp.1408187)
Supplement: (846 KB) PDF [file ehp.1408187.s001.508.pdf]

Environmental Health Perspectives doi:10.1289/ehp.1408187

Supplemental Material

Exposure to Free and Conjugated Forms of Bisphenol A and Triclosan among Pregnant Women in the MIREC Cohort

Tye E. Arbuckle, Leonora Marro, Karelyn Davis, Mandy Fisher, Pierre Ayotte, Patrick Bélanger, Pierre Dumas, Alain LeBlanc, René Bérubé, Éric Gaudreau, Gilles Provencher, Elaine M. Faustman, Eric Vigoren, Adrienne S. Ettinger, Michael Dellarco, Susan MacPherson, and William D. Fraser[7]

Table S1. Characteristics of participants in the MIREC Study (n = 1890 women) and their urine collection during the 1st trimester.

Characteristic	N	Percent
**Time of urine collection (n = 2 missing)**		
6:00 – 9:00	28	1.48
9:00 – 12:00	814	43.11
12:00 – 15:00	643	34.06
15:00 – 18:00	367	19.44
18:00 – 24:00	36	1.91
**Season of collection**		
Fall	547	28.94
Winter	456	24.13
Spring	442	23.39
Summer	445	23.54
**Time since last urine void (min.) (n = 93 missing)**		
≤ 75	488	27.16
76 - 120	597	33.22
121 – 170	266	14.80
> 170	446	24.82
**Fasting at collection (n = 25 missing)**		
No	1828	98.02
Yes	37	1.98
**Maternal age (yr.) (n = 1 missing)**		
< 25	125	6.62
25 – 29	441	23.34
30 – 34	682	36.10
≥ 35	641	33.93
**Education (n = 3 missing)**		
High school or less	168	8.90
College courses or diploma	540	28.62
University degree	1179	62.48
**Household income (n = 84 missing)**		
≤ $50,000	326	18.05
$50,001 – 100,000	754	41.75
> $100,000	726	40.20
**Country of birth**		
Canada	1537	81.32
Elsewhere	353	18.68
**Smoking status at 1st visit (n = 2 missing)**		
Current or quit during pregnancy	225	11.92
Former	521	27.59
Never	1142	60.49
**Pre-pregnancy BMI (kg/m^2) (n = 136 missing)**		
< 25 (underweight-normal)	1108	63.17
25 – 29 (overweight)	385	21.95
≥ 30 (obese)	261	14.88
**Parity (n = 2 missing)**		
0	835	44.23
1	765	40.52
≥ 2	288	15.25

Table S2. Summary of first trimester urinary concentrations of free and conjugated forms of bisphenol A (BPA) and triclosan (TCS), volumetric and specific gravity (SG) adjusted (µg equivalents/L) – Censored methods [Kaplan-Meier and Maximum Likelihood Estimation (MLE)] (n = 3 missing SG).

Contaminant	N	Kaplan-Meier Median (95% CI)[a]	MLE GM (95% CI)[b]
**BPA disulfate**	1890	ND (ND, ND)	ND (ND, ND)
SG-adjusted	1887	ND (ND, ND)	ND (ND, ND)
**BPA glucuronide**	1889	0.84 (0.78, 0.91)	0.82 (0.78, 0.87)
SG-adjusted	1886	0.87 (0.83, 0.91)	0.93 (0.89, 0.97)
**BPA free**	1885	NA	ND (ND, ND)
SG-adjusted	1882	ND (ND, ND)	ND (ND, ND)
**BPA mono-sulfate**	1885	NA	ND (ND, ND)
SG-adjusted	1882	NA	ND (ND, ND)
**Total BPA**	1879	0.89 (0.83, 0.96)	0.86 (0.81, 0.92)
SG-adjusted	1876	0.94 (0.90, 0.98)	0.97 (0.93, 1.02)
**Total TCS**	1861	8.74 (7.62, 9.86)	12.61 (11.36, 14.00)
SG-adjusted	1858	9.17 (7.90, 10.48)	14.34 (12.99, 15.84)
**TCS glucuronide**	1868	8.42 (7.29, 9.56)	12.29 (11.07, 13.64)
SG-adjusted	1865	9.03 (7.79, 10.27)	13.94 (12.63, 15.40)
**TCS free**	1882	0.06 (0.05, 0.07)	0.07 (0.06, 0.08)
SG-adjusted	1879	0.06 (0.05, 0.07)	0.08 (0.07, 0.09)
**TCS sulfate**	1890	NA	ND (ND, ND)
SG-adjusted	1887	NA	ND (ND, ND)
% free/total BPA	1879	0.83 (0.78, 0.89)	0.73 (0.66, 0.81)
% free/total TCS	1861	0.57 (0.54, 0.61)	0.61 (0.58, 0.65)

ND – Estimate below the limit of detection.

NA – Kaplan-Meier could not be produced due to high percent of censoring.

[a]Confidence Intervals computed using Greenwood's estimate of the standard error. [b]Geometric Mean and Confidence Intervals computed using the maximum likelihood method.

Table S3. Predictors of maternal urinary concentrations of total BPA (reported as μg BPA equivalents/L) including specific gravity as a covariate and based on machine readings.

Characteristic	N	% <LOD	p-value[a]	Pairwise[b]	GM (95% CI)[c]
**Maternal Age**					
<25	125	12.10	0.0012	A	1.14 (0.97, 1.34)
25-29	441	30.30		AB	0.91 (0.83, 0.99)
30-34	682	31.12		AB	0.90 (0.84, 0.97)
>=35	641	34.33		B	0.81 (0.75, 0.87)
**Parity**					
0	834	30.41	0.0505[d]		0.93 (0.87, 0.99)
1	765	31.05			0.86 (0.80, 0.92)
2+	288	31.23			0.82 (0.74, 0.92)
**Maternal Smoking Status**					
Current/quit during pregnancy	225	19.64	0.0004	A	1.09 (0.97, 1.24)
Former	521	32.30		B	0.90 (0.83, 0.97)
Never	1141	32.31		B	0.84 (0.79, 0.88)
**Maternal Education**					
High school or less	168	12.65	0.0057[d]	A	1.01 (0.87, 1.16)
College courses or diploma	540	29.00		AB	0.91 (0.84, 0.98)
University degree	1179	34.19		B	0.85 (0.81, 0.90)
**Place of Birth**					
Elsewhere	353	34.94	0.0064[d]	A	0.79 (0.72, 0.88)
Canada	1536	29.80		B	0.90 (0.86, 0.95)
**Pre-pregnancy BMI (kg/m^2)**					
< 25 (underweight-normal)	1108	33.24	0.2206		0.85 (0.80, 0.90)
25 – 29 (overweight)	384	29.84			0.91 (0.82, 1.00)
≥ 30 (obese)	261	23.37			0.94 (0.84, 1.05)
**Household Income**					
≤ $50,000	326	24.69	0.0013[d]	A	1.00 (0.90, 1.11)
$50,001 – 100,000	754	29.56		AB	0.90 (0.84, 0.96)
> $100,000	726	34.90		B	0.83 (0.78, 0.89)
**Season Urine Collected**					
Fall	547	30.83	0.0741[d]		0.86 (0.80, 0.93)
Winter	456	28.16			0.91 (0.84, 1.00)
Spring	442	33.64			0.83 (0.76, 0.90)
Summer	444	30.47			0.93 (0.85, 1.02)
**Fasting Status**					
No	1827	30.99	0.7304		0.88 (0.84, 0.92)
Yes	37	18.92			0.93 (0.69, 1.25)
**Time Since Last Urination (min.)**					
≤ 75	488	38.22	0.0045	A	0.84 (0.77, 0.91)
76 - 120	596	33.73		A	0.83 (0.77, 0.89)
121 - 170	266	27.17		B	1.03 (0.92, 1.16)
> 170	446	20.90		AB	0.93 (0.85, 1.02)

[a]p-value for overall group effect based on machine readings. [b]Group levels with the same letter indicate no significant difference using Scheffé multiple comparisons. Groups with different letters indicate significant differences in contaminant levels. [c]GM: geometric mean; 95% CI: the 95% confidence intervals for the GM were corrected for multiple comparisons using Scheffé correction. [d]ANCOVA model based on the ranks of the data (non-parametric ANCOVA). Each group is assumed to have the same slope with respect to the covariate [i.e., the interaction between the potential predictor (characteristic) and specific gravity was not significant (p>0.05)].

Table S4. Comparisons of maternal urinary concentrations of total bisphenol A (BPA)[a] (reported as µg BPA equivalents/L) at the various times of urine collection, with respect to different levels of specific gravity[b].

Time of Urine Collection	P25 of SG Differences	P25 of SG Adjusted Geometric Mean (95% CI)	P50 of SG Differences	P50 of SG[a] Adjusted Geometric Mean (95% CI)	P75 of SG Differences	P75 of SG Adjusted Geometric Mean (95% CI)
6:00 - 9:00	AB	0.31 (0.19, 0.51)	ABC	0.68 (0.48, 0.97)		1.70 (1.14, 2.55)
9:00 - 12:00	A	0.39 (0.36, 0.42)	A	0.74 (0.69, 0.79)		1.55 (1.42, 1.70)
12:00 - 15:00	A	0.40 (0.36, 0.44)	AB	0.77 (0.72, 0.83)		1.68 (1.54, 1.84)
15:00 - 18:00	B	0.61 (0.53, 0.70)	C	1.01 (0.92, 1.12)		1.83 (1.64, 2.04)
18:00 - 24:00	AB	0.77 (0.47, 1.27)	BC	1.26 (0.89, 1.79)		2.24 (1.63, 3.08)

[a]ANCOVA model based on the ranks of the data (non-parametric) model, assuming separate slopes for each of the groups of the characteristic, since the interaction between time of urine collection and SG was significant $p<0.05$. Pairwise comparisons were made at each level of SG only when the overall difference between the characteristic was significant at that level of SG. Therefore where no pairwise comparisons are made there was no significant difference between the groups of the characteristic. [b]P25 of SG is the 25th percentile of specific gravity = 1.007; P50 of SG is the 50th percentile of specific gravity = 1.013; P75 of SG is the 75th percentile of specific gravity = 1.020.

Table S5. Predictors of maternal urinary concentrations of total triclosan (reported as µg TCS equivalents/L) with specific gravity as a covariate, based on machine readings.

**Characteristic**	**N**	**% <LOD**	**p-value[a]**	**Pairwise[b]**	**GM (95% CI)[c]**
**Maternal Age**					
<25	122	0.82	0.0001[d]	A	5.38 (3.65, 7.92)
25-29	437	0.23		B	13.58 (11.07, 16.65)
30-34	672	0.89		B	13.39 (11.36, 15.78)
>=35	630	0.48		B	13.31 (11.23, 15.77)
**Maternal Smoking Status**					
Current/quit during pregnancy	220	0.45	0.0022	A	8.78 (6.57, 11.71)
Former	515	0.97		AB	11.03 (9.14, 13.32)
Never	1124	0.44		B	14.42 (12.69, 16.37)
**Maternal** Education					
High school or less	163	0.61	0.0003	A	7.78 (5.55, 10.90)
College courses or diploma	530	0.38		A	10.63 (8.83, 12.79)
University degree	1166	0.69		B	14.62 (12.90, 16.57)
**Place of Birth**					
Elsewhere	347	0.86	0.3030		11.32 (9.00, 14.25)
Canada	1514	0.53			12.94 (11.60, 14.45)
**Pre-pregnancy BMI (kg/m^2)**					
< 25 (underweight-normal)	1089	0.55	0.7080[d]		12.07 (10.61, 13.74)
25 – 29 (overweight)	383	0.26			12.55 (10.10, 15.60)
≥ 30 (obese)	258	0.39			13.09 (10.02, 17.11)
**Household Income**					
≤ $50,000	318	0.31	0.0001	A	9.68 (7.62, 12.30)
$50,001 – 100,000	743	0.40		A	10.96 (9.38, 12.81)
> $100,000	717	0.84		B	16.36 (13.95, 19.19)
**Season Urine Collected**					
Fall	540	0.19	0.3462		14.42 (12.00, 17.34)
Winter	448	0.45			12.67 (10.35, 15.51)
Spring	436	0.92			11.62 (9.47, 14.27)
Summer	437	0.92			11.59 (9.44, 14.22)
**Fasting Status**					
No	1799	0.61	0.2511		12.60 (11.39, 13.94)
Yes	37	0.00			8.31 (4.11, 16.80)
**Time of Urine Collection**					
6:00 - 9:00	28	3.57	0.3722		13.86 (6.18, 31.10)
9:00 - 12:00	797	0.63			14.15 (12.15, 16.48)
12:00 - 15:00	635	0.63			11.94 (10.08, 14.16)
15:00 - 18:00	364	0.27			11.00 (8.78, 13.78)
18:00 - 24:00	35	0.00			10.38 (5.03, 21.41)
**Time Since Last Urination (min.)**					
≤ 75	480	1.04	0.1168		10.47 (8.61, 12.73)
76 - 120	593	0.67			13.62 (11.43, 16.23)
121 - 170	262	0.38			14.56 (11.18, 18.96)
> 170	438	0.00			13.73 (11.19, 16.85)

[a]p-value for overall group effect using machine readings. [b]Group levels with the same letter indicate no significant difference using Scheffé multiple comparisons. Groups with different letters indicate significant differences in contaminant levels. [c]GM: geometric mean; 95% CI: the 95% confidence intervals for the GM was corrected for multiple comparisons using Scheffé correction. [d]ANCOVA model based on the ranks of the data (non-parametric ANCOVA). Each group is assumed to have the same slope with respect to the covariate [i.e., the interaction between the potential predictor (characteristic) and specific gravity is not significant ($p>0.05$)].

Table S6. Comparisons of maternal urinary concentrations of total triclosan (TCS)[a] (reported as µg TCS equivalents/L) at the various parity groups, with respect to different levels of specific gravity[b].

Parity	P25 of SG Differences	P25 of SG Adjusted Geometric Mean (95% CI)	P25 of SG Differences	P25 of SG Adjusted Geometric Mean (95% CI)	P25 of SG Differences	P25 of SG Adjusted Geometric Mean (95% CI)
0		6.33 (5.20, 7.70)		12.58 (10.84, 14.59)	A	28.03 (22.95, 34.23)
1		7.12 (5.76, 8.80)		11.33 (9.67, 13.27)	B	19.50 (16.09, 23.62)
2+		5.18 (3.64, 7.39)		9.69 (7.47, 12.56)	AB	20.10 (14.71, 27.45)

[a]ANCOVA model, assuming separate slopes for each of the groups of the characteristic since the interaction between parity and SG was significant ($p<0.05$). Pairwise comparisons were made at each level of SG only when the overall difference between the groups of the characteristic was significant at that level of SG. Therefore, where no pairwise comparisons were made, there was no significant difference between the groups of the characteristic. [b]P25 of SG is the 25th percentile of specific gravity = 1.007; P50 of SG is the 50th percentile of specific gravity = 1.013; P75 of SG is the 75th percentile of specific gravity = 1.020.

Table S7. Comparison of international studies that have measured total triclosan (unadjusted) in the urine of pregnant women and in women of reproductive age.

Study Population	Sample Size	Gestational Age at Collection	Triclosan Geometric Mean	Triclosan Median	Triclosan Maximum Value	Reference
California MIEEP project	77	3rd trimester	17.2 (95% CI: 10.5, 28.2) µg/L	11.1 µg/L	90th %ile: 445 µg/L	California 2013
US NHANES 2003-2004 pregnant women	86	1st – 3rd trimester	17.00 µg/L	8.2 µg/L	95th %ile: 283.0 µg/L	Woodruff et al. 2011
Case-control study of male malformations in France	191	6 – 30 wks		24.1 µg/L	95th %ile: 634 µg/L	Philippat et al. 2012
Multiethnic cohort in New York City	367	3rd trimester		11.0 µg/L	1790 µg/L	Wolff et al. 2008
INMA cohort study in Spain	120	3rd trimester		6.1 ng/mL		Casas et al. 2011
PROTECT project, Puerto Rico	105	18, 22 & 26 wks	29.9 (95% CI: 23.6, 37.9) ng/ml	26.2 ng/ml	2000 µg/L	Meeker et al. 2013
Women undergoing amniocentesis in New York City	71	18, 23 and 33 wks		6.5 µg/L at 18 wks; 15.4 at 23 wks; 16.2 at 33 wks	Maximum 95th %ile: 789 µg/L	Philippat et al. 2013
MIREC Study of Canadian women	1861	1st trimester	12.6 (95% CI: 11.4, 14.0) µg/L	8.74 µg/L	6784.3 µg/L	Our study
CHMS Cycle 2 (2009-2011) females 20-39 years of age	188		19.0 (95% CI: 11, 35) µg/L	16.0 (95% CI: 6, 27) µg/L		Health Canada 2013

Figure S1. Plots of maternal urinary concentrations of (a) Log(free BPA) vs Log(total BPA) (r = 0.42, p<0.0001) and (b) Log(%free BPA) vs Log(total BPA) (r = -0.22, p<0.0001).

S1a

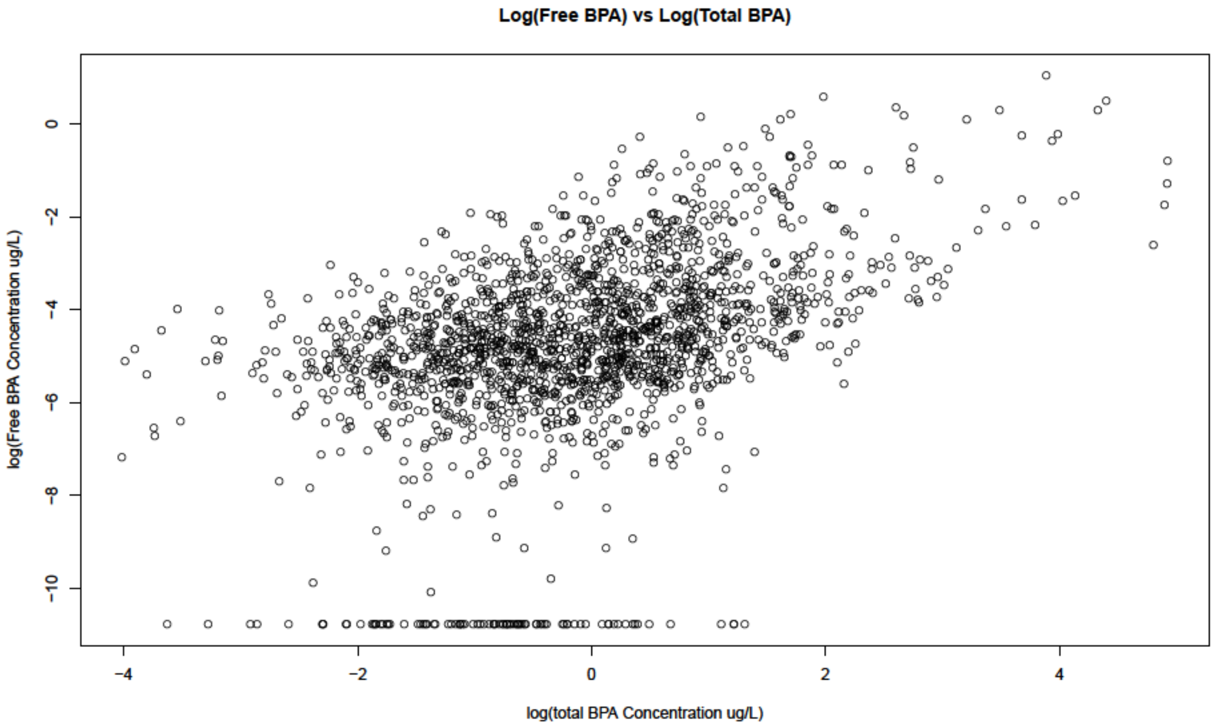

S1b

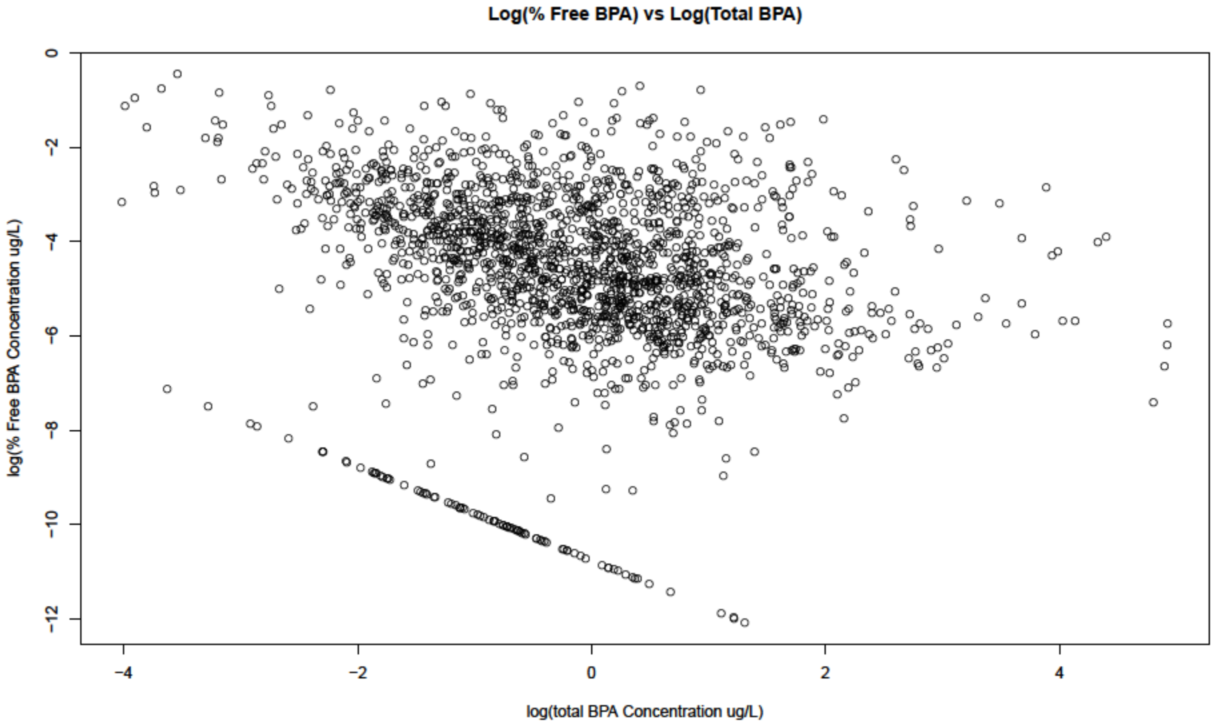
Log(% Free BPA) vs Log(Total BPA)
log(% Free BPA Concentration ug/L)
0
-2
-4
-6
-8
-10
-12
-4
-2
0
2
4
log(total BPA Concentration ug/L)

References

Braun JM, Kalkbrenner AE, Calafat AM, Yolton K, Ye X, Dietrich KN, et al. 2011. Impact of early-life bisphenol A exposure on behavior and executive function in children. Pediatrics 128(5):873-882.

California. 2013. Maternal and Infant Environmental Exposure Project (MIEEP): Chemicals in Our Bodies Project. Available: http://biomonitoring.ca.gov/projects/maternal-and-infant-environmental-exposure-project-mieep [accessed 7 November 2014].

Casas L, Fernández MF, Llop S, Guxens M, Ballester F, Olea N, et al. 2011. Urinary concentrations of phthalates and phenols in a population of Spanish pregnant women and children. Environ Int 37(5):858-866.

Casas M, Valvi D, Luque N, Ballesteros-Gomez A, Carsin AE, Fernandez MF, et al. 2013. Dietary and sociodemographic determinants of bisphenol A urine concentrations in pregnant women and children. Environ Int 56:10-18.

Health Canada. 2013. Second Report on Human Biomonitoring of Environmental Chemicals in Canada: Results of the Canadian Health Measures Survey, Cycle 2 (2009-2011), April 2013. HC Pub.: 130019. Cat.: H128-1/10-601-1E-PDF. ISBN: 978-1-100-22140-3. Available: www.healthcanada.gc.ca/biomonitoring [accessed 7 November 2014].

Hoepner LA, Whyatt RM, Just AC, Calafat AM, Perera FP, Rundle AG. 2013. Urinary concentrations of bisphenol A in an urban minority birth cohort in New York City, prenatal through age 7 years. Environ Res 122:38-44.

Lee BE, Park H, Hong YC, Ha M, Kim Y, Chang N, et al. 2013. Prenatal bisphenol A and birth outcomes: MOCEH (Mothers and Children's Environmental Health) study. Int J Hyg Environ Health Jul 11. doi:pii:S1438-4639(13)00099-0. 10.1016/j.ijheh.2013.07.005.

Meeker JD, Cantonwine DE, Rivera-González LO, Ferguson KK, Mukherjee B, Calafat AM, et al. 2013. Distribution, variability, and predictors of urinary concentrations of phenols and parabens among pregnant women in Puerto Rico. Environ Sci Technol 47(7):3439-3447.

Philippat C, Mortamais M, Chevrier C, Petit C, Calafat AM, Ye X, et al. 2012. Exposure to phthalates and phenols during pregnancy and offspring size at birth. Environ Health Perspect 120(3):464-470.

Philippat C, Wolff MS, Calafat AM, Ye X, Bausell R, Meadows M, Stone J, Slama R, Engel SM. 2013. Prenatal exposure to environmental phenols: concentrations in amniotic fluid and variability in urinary concentrations during pregnancy. Environ Health Perspect 121(10):1225-1231.

Wolff MS, Engel SM, Berkowitz GS, Ye X, Silva MJ, Zhu C, et al. 2008. Prenatal phenol and phthalate exposures and birth outcomes. Environ Health Perspect 116(8):1092-1097.

Woodruff TJ, Zota AR, Schwartz JM. 2011. Environmental chemicals in pregnant women in the United States: NHANES 2003-2004. Environ Health Perspect 119(6):878-885.
